# WRIST-WORN TRI-AXIAL ACCELEROMETER VALIDATION IN YOUNG, MIDDLE-AGED, AND OLDER ADULTS

**DOI:** 10.1093/geroni/igz038.1210

**Published:** 2019-11-08

**Authors:** Todd M Manini, Santiago Saldana, Duane Corbett, Amal A Wanigatunga, Eduardo Navarro, Mamoun T Mardini, Ramon Casanova

**Affiliations:** 1 University of Florida, Gainesville, Florida, United States; 2 Wake Forest University, Winston-Salem, North Carolina, United States; 3 Johns Hopkins Bloomberg School of Public Health, Baltimore, Maryland, United States

## Abstract

Purpose: This study evaluated wrist-worn accelerometers for estimating metabolic intensity and classifying activity types across a wide age spectrum. Methods: Participants (n=141, 67% women, aged 20-89 yrs) performed a battery of 31 common daily activities (e.g. washing dishes, walking) in a standardized laboratory setting. A tri-axial accelerometer was worn on the right wrist during each activity whiel a portable metabolic unit was used to measure oxygen consumption (ml/kg/min), which was converted into metabolic equivalents (METs). Random forest analyses estimated metabolic intensity and classified activity type based on seven data features. Resulting estimates were cross-evaluated on a separate sample of 16 participants who performed a sub-set of activities in their home. Results: In the laboratory setting, mean differences between measured and predicted MET value for sedentary (0.36), lifestyle (0.02) and locomotor (0.30) activities were low, but the 95% limits of agreement ranges were relatively large (+/-1.0, +/-1.8, +/-3.1, respectively). Data features were 85%, 88%, and 71% accurate for identifying sedentary, lifestyle and locomotor activities. Prediction equations had an overall mean difference of 0.19 METs (95% limits of agreement = -1.3 to 1.7) when activities were performed at home. Conclusion: Data features extracted from a wrist worn tri-axial accelerometer provide a moderate-to-high group estimate of metabolic intensity and had modest accuracy in identifying activity types across a variety of daily activities. However, significant between person variations were evident. Additional work is needed to refine wrist-worn accelerometers for estimating physical activity type, intensity, duration and frequency across the age spectrum.

